# A case of refractory chylothorax due to an unenclosed esophageal hiatus after subtotal esophagectomy treated with lipiodol lymphangiography

**DOI:** 10.1186/s40792-024-02019-0

**Published:** 2024-09-11

**Authors:** Koji Kaneda, Takeshi Miwa, Tomoyuki Okumura, Yoshihisa Numata, Mina Fukasawa, Toru Watanabe, Isaya Hashimoto, Norihito Naruto, Tsutomu Fujii

**Affiliations:** 1 Department of Surgery and Science, Faculty of Medicine, Academic Assembly, University of Toyama, 2630 Sugitani, Toyama, 930-0194 Japan; 2 Department of Radiology, University of Toyama, 2630 Sugitani, Toyama, 930-0194 Japan

**Keywords:** Chylothorax, Esophagectomy, Lipiodol lymphangiography, Esophageal hiatus, Chylous ascites

## Abstract

**Background:**

Chylothorax, a rare but serious complication following esophagectomy, can lead to dehydration, malnutrition, and even mortality. Surgical intervention is considered when conservative treatment is ineffective; however, in some refractory cases, the cause of chylothorax remains unclear. We report a case of refractory chylothorax caused by abdominal chyle leakage into the pleural space via an unenclosed esophageal hiatus.

**Case presentation:**

A 66-year-old man was diagnosed with advanced esophageal squamous cell carcinoma. The patient underwent robot-assisted thoracoscopic subtotal esophagectomy in the prone position with retrosternal gastric tube reconstruction following neoadjuvant chemotherapy. The thoracic duct was ligated and resected because of tumor invasion. Chylothorax and chylous ascites were observed 2 weeks after surgery but did not improve despite conservative management with medications and drainage. Lymphoscintigraphy through the inguinal lymph node showed tracer accumulation in the fluid in both the abdominal and pleural spaces. Lipiodol lymphangiography revealed abdominal lymphoid leakage, but no leakage was detected from the thoracic duct or mediastinum. We considered that the chylothorax was caused by chylous ascites flowing into the pleural space via an unenclosed esophageal hiatus, and we performed surgical intervention. Laparotomy revealed abdominal chyle leakage and a fistula at the esophageal hiatus with the inflow of ascites into the thoracic cavity. Lipiodol lymphangiography was additionally performed for treating abdominal lymphorrhea after surgery, and resulted in the improvement of the chylothorax and ascites. The patient was discharged with no recurrence of chylothorax or chylous ascites.

**Conclusions:**

Refractory chylothorax can occur due to chylous ascites flowing into the pleural space via an unenclosed esophageal hiatus. When the site of chylothorax leakage is unclear, the possibility of inflowing chylous ascites via the unenclosed esophageal hiatus should be explored. Esophageal hiatus closure and lipiodol lymphangiography could be effective in treating refractory chylothorax of unknown cause after esophagectomy.

## Introduction

Chylothorax, a rare but severe complication following esophagectomy, can lead to dehydration, malnutrition, and even mortality [1]. The primary treatment for this complication is conservative therapy, including fasting, total parenteral nutrition, drug therapy, and pleural drainage. Surgical treatment is considered when conservative therapy is ineffective. Recently, lipiodol lymphangiography has been reported to be an effective treatment for chylothorax because it can help in both the detection of lymphorrhea sites and the resolution of leakage [2]. Chylothorax is generally caused by thoracic duct injury, regardless of whether the duct is preserved or resected with the esophagus [3]. However, in some cases, the cause of chylothorax remains unclear. Here, we report a case of refractory chylothorax caused by flowing chylous ascites into the pleural space via the unenclosed esophageal hiatus. The chylothorax was successfully treated with esophageal hiatus closure and lipiodol lymphangiography.

## Case presentation

**Fig. 1:**
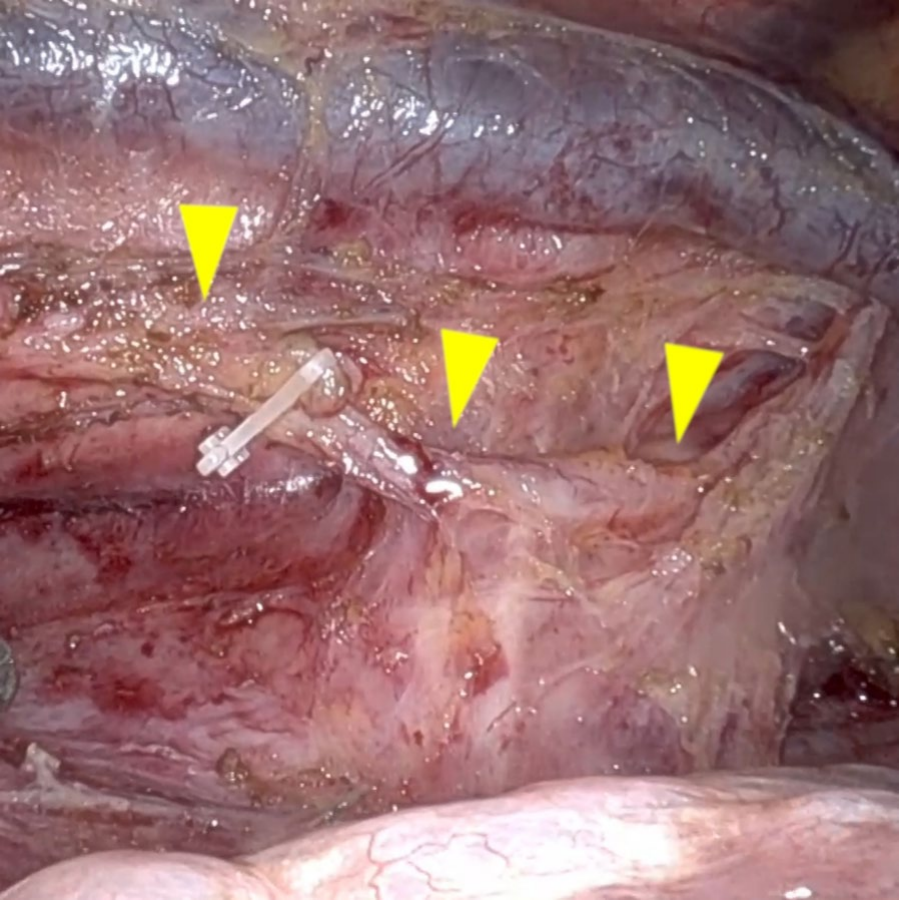
Intraoperative findings of thoracoscopic esophagectomy. The thoracic duct (arrow head) was invaded by the tumor and resected after clipping

**Fig. 2:**
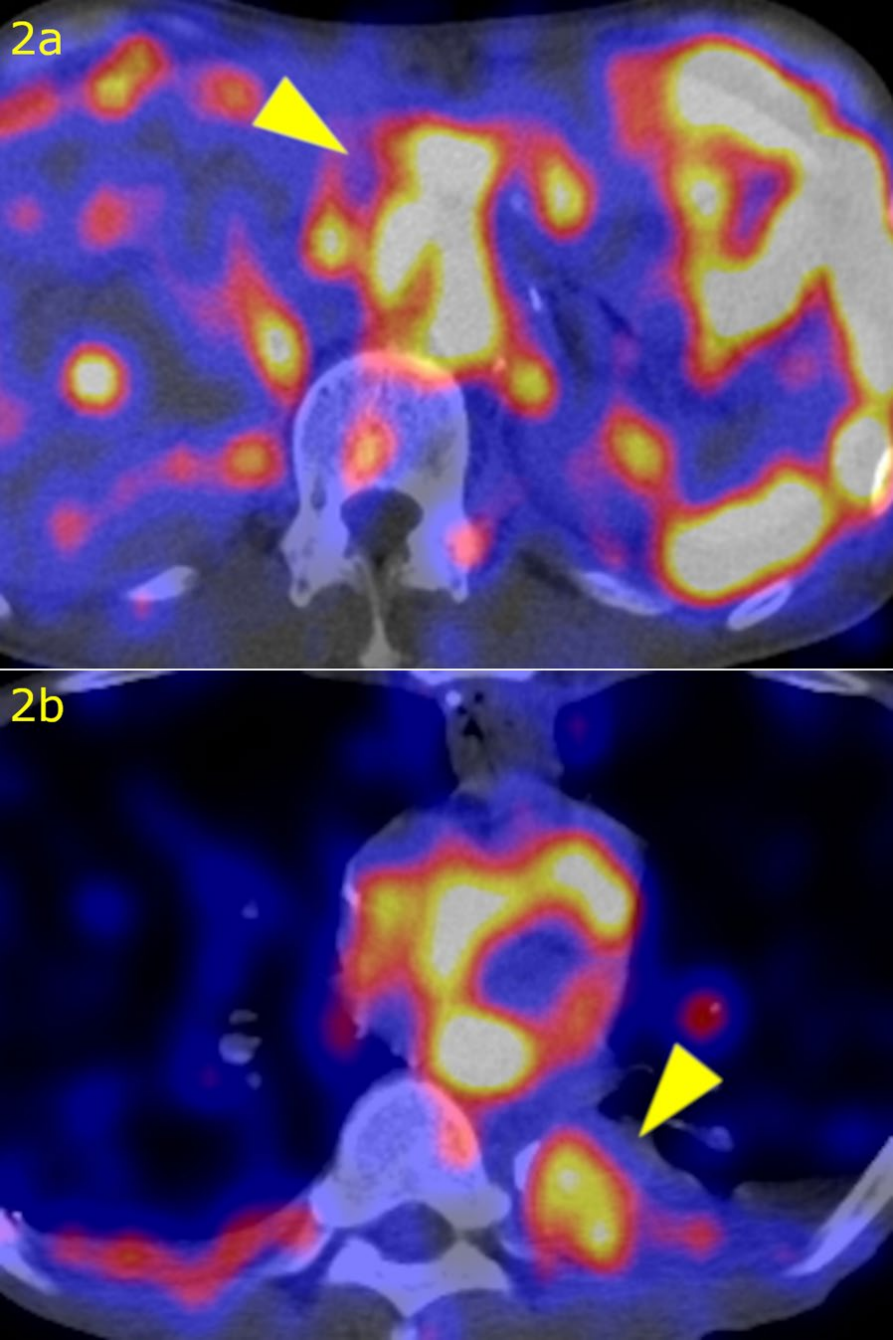
Lymphoscintigraphy. Tracer accumulation (arrow head) in ascites (**a**) and in pleural effusion fluid (**b**)

**Fig. 3:**
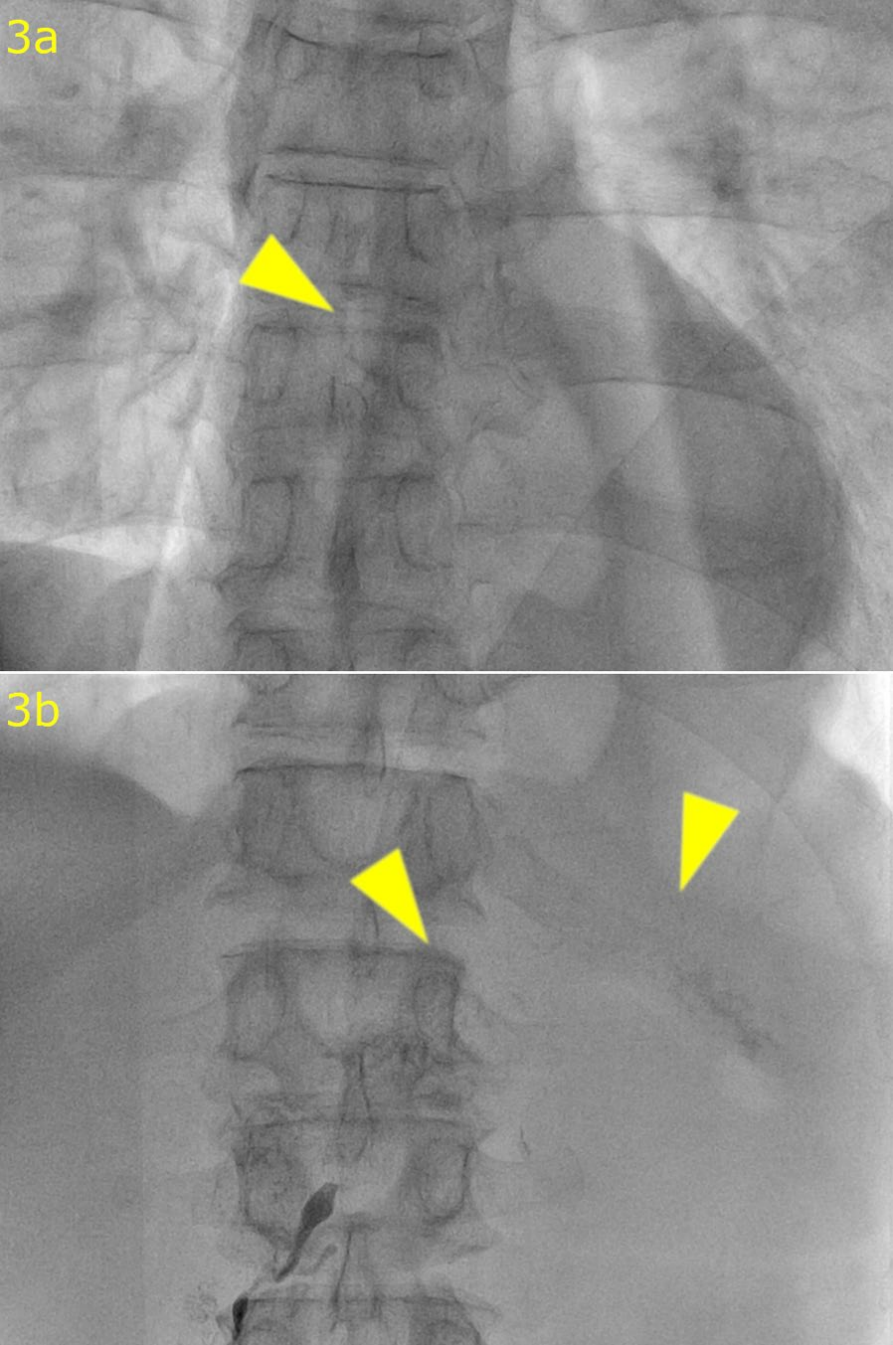
Lipiodol lymphangiography. **a** No leakage at the mediastinum (arrow head). **b** Leakage to the upper abdominal space (arrow head)

**Fig. 4:**
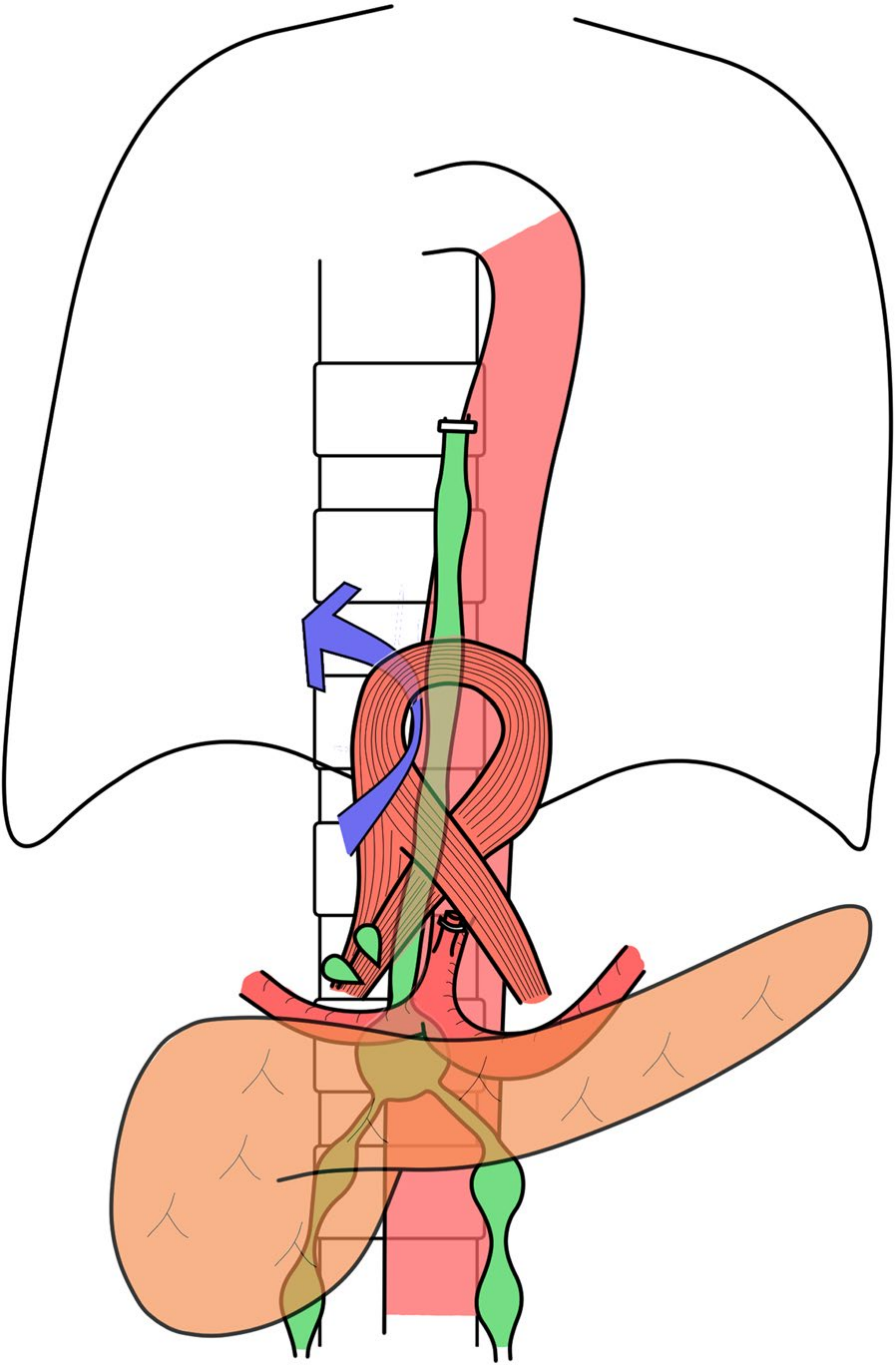
Schematic for refractory chylothorax caused by the inflow of chylous ascites into the lower-pressure thoracic cavity via an unenclosed esophageal hiatus

**Fig. 5:**
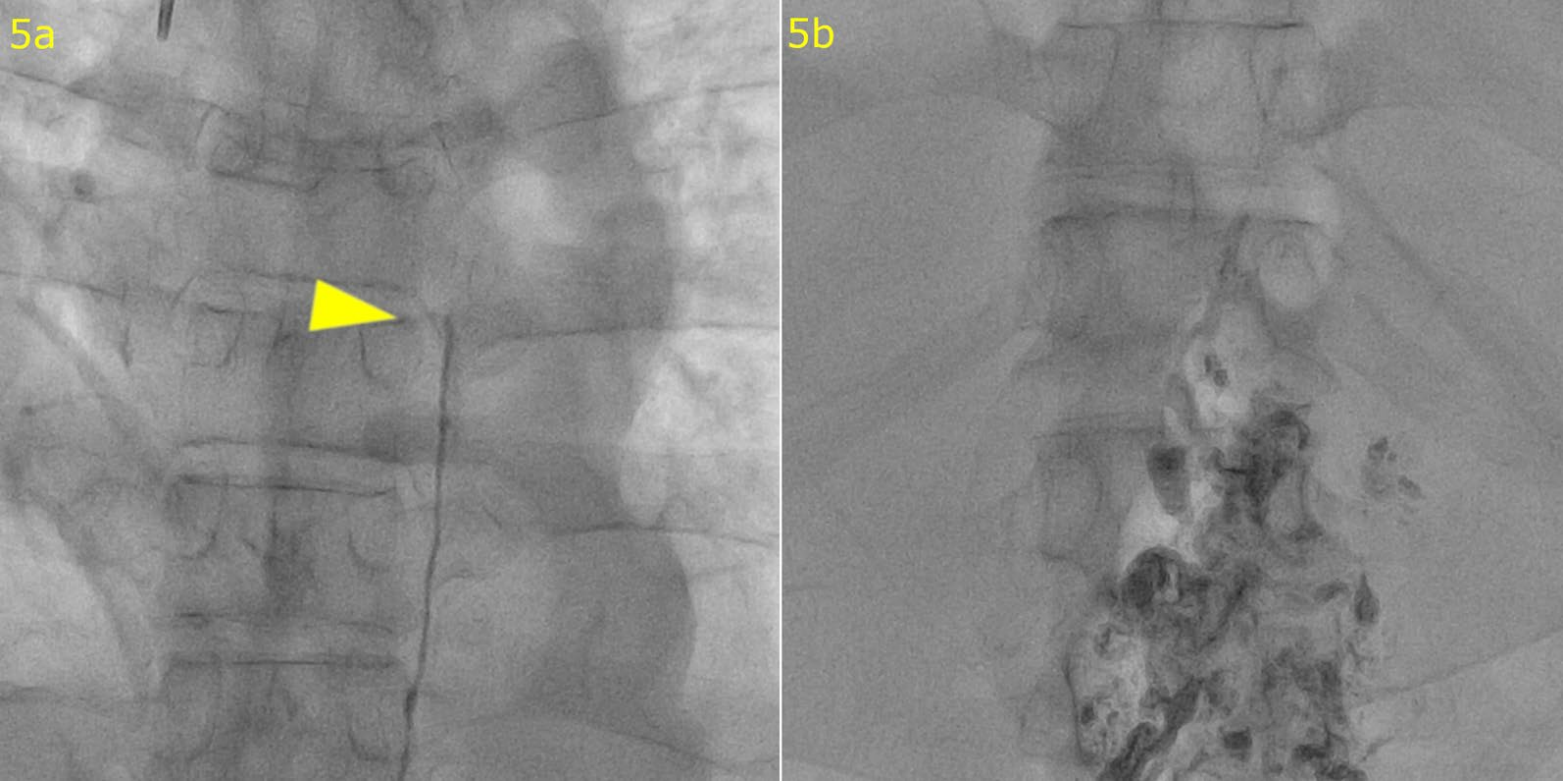
Third lipiodol lymphangiography. **a** No leakage from the thoracic duct (arrow head). **b** Improved leakage to the upper abdominal space

A 66-year-old man who suffered from dysphasia was diagnosed with Stage IIIC (cT4aN1M0) middle thoracic esophageal squamous cell carcinoma according to the 7th edition of the Union for International Cancer Control TNM staging system [4]. He received 3 cycles of docetaxel, cisplatin, and 5-fluorouracil neoadjuvant chemotherapy [5, 6] and underwent robot-assisted thoracoscopic esophagectomy in the prone position with laparoscopy-assisted retrosternal gastric tube reconstruction. During surgery, the thoracic duct was ligated and resected because of invasion by the tumor, and the left pleural cavity was opened (Fig. 1). The thoracic drainage tube was removed on postoperative day (POD) 5. Chylous ascites leakage was observed from the feeding tube fistula on POD 15 and was reduced after conservative fasting management. Bilateral pleural effusions were detected on POD 25 and treated by the placement of a drainage tube. Effusions were improved and the drainage tube was removed on POD 28. The patient was discharged on POD 32 with little pleural effusion. The patient underwent drainage of the pleural fluid as an outpatient on POD 46, but the pleural fluid increased again, leading to readmission on POD 50. Blood tests and CT scans of the chest and abdomen did not reveal any infection, thrombus, liver dysfunction, or portal vein dilatation. The pleural effusion was milky white and high in triglycerides, and the patient was diagnosed with chylothorax. We treated the patient with thoracic drainage, total parenteral nutrition, and continuous octreotide and etilefrine administration, although the pleural effusion volume remained in the range of 1500–2000 ml/day. Lymphoscintigraphy revealed tracer accumulation in the ascites and pleural effusion fluid (Fig. 2). Lipiodol lymphangiography through right inguinal lymph node puncture revealed lipiodol leakage at the upper abdominal space but no leakage from the thoracic duct or mediastinum (Fig. 3). Based on these findings, we determined that the cause of refractory chylothorax was the continuous inflow of chylous ascites into the lower-pressure thoracic cavity via an unenclosed esophageal hiatus (Fig. 4). We performed esophageal hiatus closure 61 days after esophagectomy. After laparotomy, there was chylous leakage around the suprapancreatic lymph node dissection site. A 5 mm fistula at the esophageal hiatus and inflow of chylous ascites were observed, so the hiatal fistula was closed with a non-absorbable suture. Polyglycolic acid sheets with fibrin glue were placed to cover the closed fistula. Although lymphatic leakage from the upper pancreatic region was noted, the specific lymphatic fistula could not be identified. A drainage tube was inserted under the left diaphragm. Second and third lipiodol lymphangiography was performed to treat the remaining abdominal lymphorrhea, and the pleura and ascites were stopped (Fig. 5). No leakage from the thoracic duct or mediastinum was observed throughout the three times of lymphangiography. After receiving nutritional support, rehabilitation, and treatment for temporary lower extremity edema, the patient was discharged without pleural effusion or ascites; 1 year after surgery, he was alive with no recurrence of carcinoma, pleural effusion, or ascites.

## Discussion

Chylothorax is a relatively rare complication after esophagectomy, with reported incidences ranging from 0.9 to 9.0% [1]. Early treatment is important for the depletion of lymphatic fluid, which can cause dehydration, electrolyte abnormalities, and malnutrition [7]. Chylothorax reportedly increases the incidence of severe complications and mortality [8]. Treatment for chylothorax can be divided into conservative and surgical approaches. Conservative treatment includes fasting, total parenteral nutrition, drug therapy (octreotide, etilefrine), and pleural effusion drainage. Surgical treatment is considered when conservative therapy is ineffective [8]. Postoperative chylothorax often occurs due to thoracic duct injury, but other causes are often unclear [9]. In this case, refractory chylothorax occurred due to the flow of chylous ascites into the pleural space via the unenclosed esophageal hiatus. To treat chylothorax and ascites, this patient underwent esophageal hiatus closure to prevent inflow to the pleural space and lipiodol lymphangiography to suppress abdominal chyle leakage.

Chylothorax is often caused by injuries to the main thoracic duct or large tributaries during thoracic surgery [9]. The site of chylous leakage can be diagnosed by lymphoscintigraphy or lymphangiography. In this patient, lymphoscintigraphy revealed radioisotope collection in both pleural and abdominal lymphoid fluid, but lymphangiography revealed only abdominal lymphoid leakage, not leakage from the thoracic duct or mediastinum. Based on these findings, we determined that the chylous ascites flowed into the pleural space via the unenclosed esophageal hiatus. The thoracic cavity is a lower-pressure cavity than the abdominal cavity and could help sustain the inflow of ascites to the thoracic cavity. During retrosternal reconstruction after subtotal esophagectomy, the esophageal hiatus may not remain unenclosed; this should not matter in most cases, but when large ascites is complicated, the unenclosed hiatus could serve as an escape route to the thoracic cavity and prevent healing of the ascites. We think that closing the esophageal hiatus is essential to prevent not only hiatal hernia but also flowing ascites into the pleural space through the hiatus, when we perform retrosternal gastric tube reconstruction following subtotal esophagectomy. There are some reports of postesophagectomy chylothorax without leakage from the thoracic duct, which was improved after lipiodol lymphangiography [10, 11]. Although not mentioned in the reports, it is possible that the etiology involved the inflow of chylous ascites to the pleural cavity. Although the inflow of chylous ascites via the unenclosed esophageal hiatus has not been reported to our knowledge, it should be considered a possible cause of refractory chylothorax.

It has been shown that lipiodol lymphangiography are effective for refractory chylothorax in this patient. When the site of chylous leakage, such as the thoracic duct, is identified, surgical closure of the leakage site is effective. However, it should be difficult to treat when the leakage site cannot be identified. Lipiodol lymphangiography has been reported to be highly effective for detecting the small leakage sites [9] and for the treatment of chylous ascites following abdominal lymph node dissection [12]. Repeated lymphangiography had been shown to enhance treatment effectiveness [13]. The mechanism is reported to be the induction of inflammation by leaked lipiodol, which induces surrounding granulation tissue formation, ultimately closing the site of leakage [9, 14].

Although this treatment is primarily indicated for abdominal chylous leakage, it should be effective for decreasing the amount of thoracic lymphatic fluid if there is an inflow of ascites to the thoracic cavity. In this case, only abdominal chylous leakage, not leakage from the thoracic duct, was detected, although chylothorax was improved after lipiodol lymphangiography. This finding indicated that the abdominal lymphatic fistula contributed to aggravating the chylothorax. Furthermore, closure of the inflow channel is also considered important. Closure of the esophageal hiatus increases intra-abdominal pressure by decreasing the escape of chylous ascites into the thoracic cavity, which could improve abdominal chylous leakage. Therefore, esophageal hiatus closure was necessary for decreasing both chylothorax and chylous ascites. The present case demonstrates refractory chylothorax, in which the site of chylous leakage that had not been detected in the thoracic duct or mediastinum, could be effectively treated through a combination of esophageal hiatus closure and repeated lipiodol lymphangiography.

Clinically, this case presents as refractory chylothorax; however, the essential pathology is chylothorax due to the influx of chylous ascites, accompanied by severe refractory chylous ascites leakage. There were not observed findings contributing to the increase in chylous ascites such as liver dysfunction, portal or venous thrombosis, intra-abdominal malignancy, or infection foci. It is considered that the chylous leakage from the supra-pancreatic lymph node dissection site worsened due to increased lymphatic pressure following thoracic duct ligation. Generally, chylous leakage after abdominal surgery is treatable conservatively [15], which is attributed to the normal lymphatic pressure (no ligation of thoracic duct) and the closed intra-abdominal pressure. In this case, the factors contributing to the refractory ascites include the excessive leakage due to increased lymphatic pressure from thoracic duct ligation and the lack of elevated intra-abdominal pressure due to the patency of the esophageal hiatus. This is evident from the fact that a hiatal closure surgery and three times of lipiodol lymphangiography were required before the ascites leakage stopped.

## Conclusion

In this case report, we present a rare case of refractory chylothorax caused by chylous ascites flowing into the pleural space via an unenclosed esophageal hiatus after subtotal esophagectomy. We demonstrated the effectiveness of esophageal hiatus closure and repeated lipiodol lymphangiography for treating this condition. When the site of chylous leakage is unknown and is not detected around the thoracic duct or mediastinum, it is important to consider an unclosed esophageal hiatus and abdominal chyle leakage. Lipiodol lymphangiography is crucial for diagnosing the mechanism and treating refractory chylothorax.

## Data Availability

The datasets of this case report are available from the corresponding author upon reasonable request.

## Abbreviations

POD: Postoperative day
CT: Computed tomography

## References

[1] Kranzfelder M, Gertler R, Hapfelmeier A, Friess H, Feith M. Chylothorax after esophagectomy for cancer: impact of the surgical approach and neoadjuvant treatment: systematic review and institutional analysis. Surg Endosc. 2013;27:3530–8. 23708712 10.1007/s00464-013-2991-7

[2] Matsumoto T, Yamagami T, Kato T, Hirota T, Yoshimatsu R, Masunami T, et al. The effectiveness of lymphangiography as a treatment method for various chyle leakages. Br J Radiol. 2009;82:286–90. 19029221 10.1259/bjr/64849421

[3] Mine S, Udagawa H, Kinoshita Y, Makuuchi R. Post-esophagectomy chylous leakage from a duplicated left-sided thoracic duct ligated successfully with left-sided video-assisted thoracoscopic surgery. Interact Cardiovasc Thorac Surg. 2008;7:1186–8. 18782784 10.1510/icvts.2008.185306

[4] Brierley J, Gospodarowicz MK, Wittekind CH. TNM classification of malignant tumours. 8th ed. Chichester: Wiley; 2017. p. 57–62.

[5] Ozawa S, Uchi Y, Ando T, Hayashi K, Aoki T. Essential updates 2020/2021: recent topics in surgery and perioperative therapy for esophageal cancer. Ann Gastroenterol Surg. 2023;7:346–57. 37152779 10.1002/ags3.12657 PMC10154818

[6] Wakita A, Motoyama S, Sato Y, Nagaki Y, Fujita H, Kemuriyama K, et al. Preoperative neoadjuvant chemoradiotherapy provides borderline resectable thoracic esophageal cancer with equivalent treatment results as clinically T3 thoracic esophageal cancer. Ann Gastroenterol Surg. 2023;7(6):904–12. 37927919 10.1002/ags3.12706 PMC10623951

[7] Yamamoto M, Miyata H, Yamasaki M, Maeda N, Miyazaki Y, Takahashi T, et al. Chylothorax after esophagectomy cured by intranodal lymphangiography: a case report. Anticancer Res. 2015;35:891–5. 25667471

[8] Shah RD, Luketich JD, Schuchert MJ, Christie NA, Pennathur A, Landreneau RJ, et al. Postesophagectomy chylothorax: incidence, risk factors, and outcomes. Ann Thorac Surg. 2012;93:897–903 (**discussion 903–4**). 22245587 10.1016/j.athoracsur.2011.10.060 PMC3430511

[9] Kawasaki R, Sugimoto K, Fujii M, Miyamoto N, Okada T, Yamaguchi M, et al. Therapeutic effectiveness of diagnostic lymphangiography for refractory postoperative chylothorax and chylous ascites: correlation with radiologic findings and preceding medical treatment. Am J Roentgenol. 2013;201:659–66. 23971461 10.2214/AJR.12.10008

[10] Manipadam JM, Kumar CS, Antony R, Yadav A, Ramesh H. An unusual cause of chylothorax after esophagectomy. Surg J. 2020;6:e157–9. 10.1055/s-0040-1713417 PMC7487323 32939399

[11] Nakano Y, Shibasaki S, Goto A, Umeki Y, Nakauchi M, Nakamura K, et al. A successful case of treatment by lymphangiography for chylothorax after robotic esophagectomy—a case of report. Gan To Kagaku Ryoho. 2021;48:1862–4. 35045429

[12] Sommer CM, Pieper CC, Itkin M, Nadolski GJ, Hur S, Kim J, et al. Conventional lymphangiography (CL) in the management of postoperative lymphatic leakage (PLL): a systematic review. Rofo. 2020;192:1025–35. 32215900 10.1055/a-1131-7889

[13] Kariya S, Nakatani M, Yoshida R, Ueno Y, Komemushi A, Tanigawa N. Repeated intranodal lymphangiography for the treatment of lymphatic leakage. Lymphology. 2015;48:59–63. 26714370

[14] Pabst TS 3rd, McIntyre KE Jr, Schilling JD, Hunter GC, Bernhard VM. Management of chyloperitoneum after abdominal aortic surgery. Am J Surg. 1993;166:194–8. 8352415 10.1016/S0002-9610(05)81055-4

[15] Russell T, Tanase A, Bowles M, Briggs C, Kanwar A, Stell D, et al. Chyle leak following pancreatico-duodenectomy: a tertiary hepatopancreaticobiliary unit’s experience and a proposed management algorithm. ANZ J Surg. 2021;91:355–60. 33459512 10.1111/ans.16535

